# Presentation of Neurological Rosai–Dorfman Disease Over 12 Years: A Case Report

**DOI:** 10.1155/crnm/8933434

**Published:** 2026-07-26

**Authors:** Sam E. Gary, Serdar Akkol, Harshadayani J. Kumar, Christina Appin, Xiaosi Han

**Affiliations:** ^1^ Medical Scientist Training Program, University of Alabama at Birmingham, Birmingham, Alabama, USA, uab.edu; ^2^ Department of Neurology, University of Alabama at Birmingham, Birmingham, Alabama, USA, uab.edu; ^3^ Department of Pediatrics, University of Alabama at Birmingham, Birmingham, Alabama, USA, uab.edu; ^4^ Department of Pathology, University of Alabama at Birmingham, Birmingham, Alabama, USA, uab.edu

**Keywords:** central nervous system, extranodal disease, histiocytosis, pachymeningeal enhancement, rare disease

## Abstract

Rosai–Dorfman disease (RDD) is a rare, non‐Langerhans cell histiocytosis that classically presents with cervical lymphadenopathy. Neurological involvement occurs in less than 5% of cases and is often associated with extra‐axial lesions. Diagnosis of RDD in the setting of neurological deficits is challenging, as RDD often mimics meningioma and neurosarcoidosis on neuroimaging. Herein, we describe a case report of a 50‐year‐old African American man who was previously treated with radiation for presumed meningioma of the brainstem 12 years prior to the current presentation. The brainstem lesion progressed to include the pachymeninges despite radiation, and the patient developed papilledema and involvement of the bilateral cochlea. Lack of response to radiation, involvement of the pachymeninges, and development of papilledema and bilateral hearing loss led to an updated diagnosis of neurosarcoidosis. On current presentation, the patient was brought to the emergency department for altered mental status, seizures, and left‐sided hemiparesis. Initial workup was negative for stroke, meningitis, and toxic or metabolic encephalopathies. Long‐term electroencephalography confirmed seizures originating from the right frontal lobe, which was controlled with antiseizure medications. Magnetic resonance imaging revealed worsening diffuse brainstem and pachymeningeal enhancement of the bilateral cerebral convexities, suggesting progression of the disease. Workup for systemic malignancy revealed a hypodense soft tissue mass encasing the left kidney, raising concern for metastatic disease. Biopsy of the meninges and the renal mass confirmed RDD with multiorgan involvement. Diagnosis of RDD in this patient marks the longest duration from onset to diagnosis ever reported, indicating the need for tissue diagnostics in uncertain cases.

## 1. Background

Rosai–Dorfman disease (RDD) is a rare, typically self‐limiting non‐Langerhans cell histiocytosis. Its clinical recognition improved following the implementation of specific diagnostic criteria [1]. RDD may present in isolation or in association with autoimmune diseases, hereditary conditions, or malignancies. RDD classically causes bilateral cervical lymphadenopathy [2]. However, extranodal RDD may be present at higher rates than previously thought, including as the sole manifestation in as many as 67% of patients [3]. Central nervous system (CNS) involvement remains uncommon, occurring in less than 5% of patients [4].

## 2. Case Presentation

**FIGURE 1:**
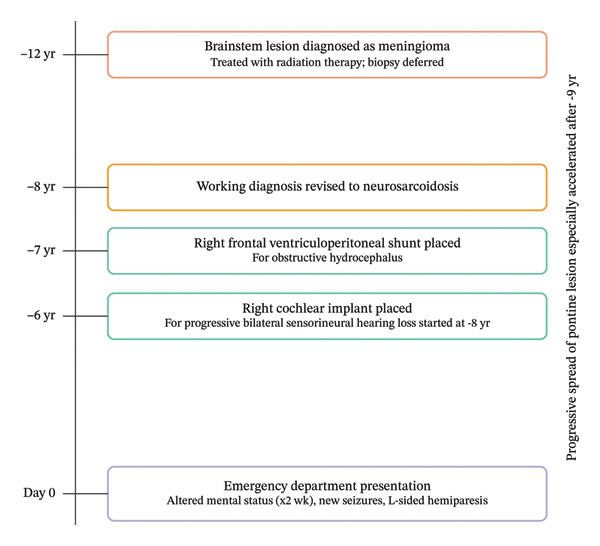
Timeline of the patient’s disease progression.

**FIGURE 2:**
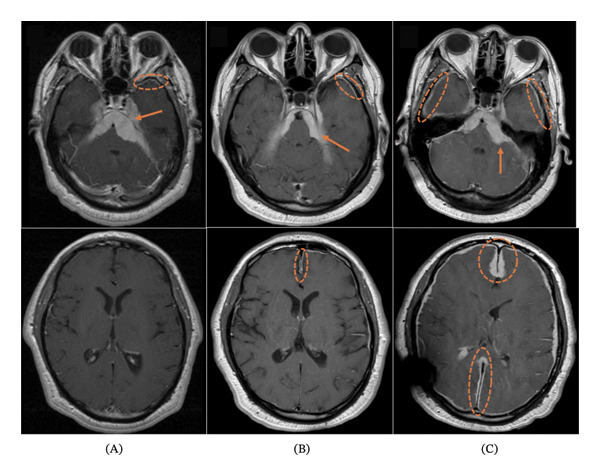
Postcontrast T1‐weighted MR of the patient’s intracranial lesions at the time of presumed diagnosis of meningioma 12 years prior to presentation (A), postradiation and presumed diagnosis of neurosarcoidosis 7 years prior to presentation (B), and at the time of diagnosis with RDD (C). Arrows indicate retroclival/prepontine lesion that was targeted for radiation. Dashed ovals indicate areas of meningeal spread.

The patient was a 50‐year‐old African American man who was brought to the emergency department for altered mental status that worsened over the course of two weeks, alongside new‐onset seizures and left‐sided hemiparesis that began on the day of arrival. He had a past medical history of bilateral hearing loss (managed with a right cochlear implant), obstructive hydrocephalus treated with a ventriculoperitoneal shunt, and brainstem lesion with postcontrast T1 enhancement in the retroclival prepontine area. This lesion was first diagnosed empirically as meningioma and treated with 50.4 Gy of fractionated radiation 12 years prior to the current presentation (Figure 1). Due to location of the lesion in the brainstem (Figure 2A), biopsy was deferred at that time. The patient’s hearing loss progressively worsened over several years, and the pontine brain lesions progressively spread throughout the meninges (Figure 2B). Therefore, a multidisciplinary team including neurosurgery, otolaryngology, and radiation oncology updated the working diagnosis to be neurosarcoidosis.

Upon arrival at the emergency room, the patient was initially evaluated for acute stroke due to left hemiparesis. His last known well was the day before admission. His temperature was 102.0 F, blood pressure was 151/100 mmHg, and blood glucose was 209 mg/dL. He was awake and alert but confused and yelling with mild dysarthria. He had a right gaze deviation that reached midline, mild left facial weakness, and absence of pain sensation and movement in his left hemibody. All other examination findings were normal. NIH Stroke Scale was 17, though the assessment was impacted by his hearing impairment. Noncontrast computed tomography (CT) and CT angiography ruled out stroke. Thus, no stroke intervention was planned. Magnetic resonance imaging (MRI) demonstrated diffuse postcontrast pachymeningeal enhancement along the bilateral cerebral convexities, suggesting progression of neurosarcoidosis (Figure 2C) along with a new, right temporal subcutaneous lesion. Long‐term electroencephalography (LTEEG) captured focal seizures originating from the right hemisphere which was treated with levetiracetam 2000 mg twice daily and lacosamide 150 mg twice daily. No further seizures were identified, and LTEEG was discontinued after 3 days. Lacosamide was later changed to oxcarbazepine 600 mg twice daily due to concern for oversedation. On admission, electrolytes were normal, but white blood cell count, lactic acid, angiotensin‐converting enzyme, and 1,25‐cholecalciferol were elevated. Urine drug screen, infectious workup, and autoimmune markers (e.g. ANA, anti‐dsDNA, SSA/B, c‐ANCA, p‐ANCA, C3, C4, and MPO) were negative. Lumbar puncture was deferred due to concern for increased intracranial pressure.

**FIGURE 3:**
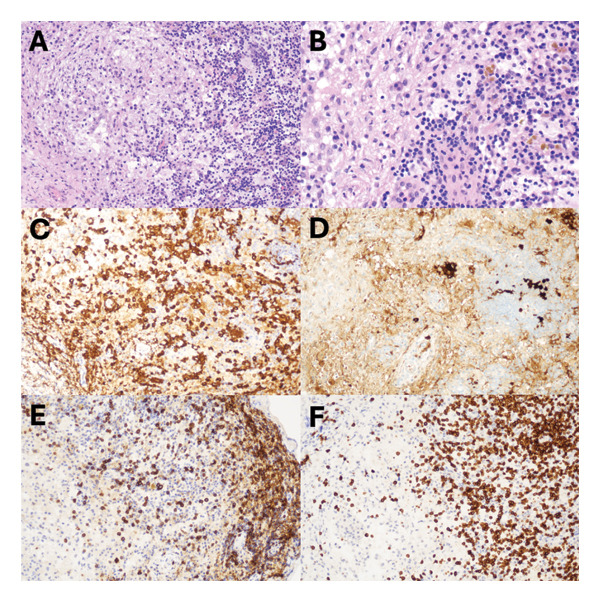
Hematoxylin and eosin staining showing active, infiltrative macrophages, lymphocytes, and plasma cells at 20x magnification (A) and emperipolesis at 40x magnification (B). Immunohistochemistry staining at 20x magnification of CD163 (C), S100 (D), CD20 (E), and CD3 (F).

The patient’s hemiparesis improved over the course of 3–4 days and was attributed to Todd’s paralysis due to the absence of acute infarcts or hemorrhages. The patient was initially treated for neurosarcoidosis with dexamethasone 4 mg every 6 h, which was later titrated down to twice daily with gradual improvement in mental status and motor strength. Because of his improvement with steroids, diffuse brainstem and meningeal enhancement, and subacute altered mental status and hemiparesis, the patient was evaluated for systemic malignancy. The patient underwent CT of his abdomen and pelvis, which identified a hypodense soft tissue mass encasing the left kidney. Biopsies of the left frontal meningeal and perirenal soft tissue both demonstrated histiocytic infiltrates with plasma cells, reactive T cells, and emperipolesis (Figure 3A,B). Immunohistochemistry staining was positive for CD163, S100, CD20, and CD3 (Figure 3C–F), Factor XIIIA, cyclin D1, and negative for ALK, BRAF, CD1a, and GMS. A definitive diagnosis of RDD was therefore made.

The patient received rituximab 800 mg with some improvement, followed by cobimetinib, a MEK inhibitor recently FDA‐approved for treatment of RDD, which improves outcomes in patients with KRAS or MEK alterations [5]. However, one day after starting cobimetinib, the patient’s confusion and agitation worsened. About 1 week later, following an unwitnessed fall and seizures, the patient suffered pulseless electrical activity. The patient was resuscitated, but he developed a saddle pulmonary embolism and obstructive shock. Per family request, the patient was transitioned to comfort care and expired shortly thereafter.

## 3. Discussion and Conclusions

Patients with RDD can present with contrast‐enhancing brainstem and extra‐axial lesions that mimic meningioma, and they can present with pachymeningeal contrast enhancement, elevated inflammatory markers, and peripheral masses that mimic sarcoidosis. Herein, we describe a 50‐year‐old patient whose disease remained inaccurately diagnosed for at least 12 years. He was originally diagnosed with meningioma, though he did not receive confirmation with biopsy due to the location in the prepontine area. The subsequent progression despite radiation therapy suggested a more complex etiology; thus, the working diagnosis was updated to neurosarcoidosis. While secondary development of RDD cannot be ruled out, the sustained appearance and slow progression of the brainstem lesion and pachymeningeal enhancement, combined with the multisystemic brain, skin, and renal lesions, suggest that this patient likely had RDD rather than meningioma or neurosarcoidosis from the outset. Prognosis was guarded in this patient due to disease burden, multisystemic extranodal disease, and unknown genetic makeup.

A primary learning point in this case report is that diagnosis of RDD can be delayed due to its mimicry of meningioma, neurosarcoidosis (or other rheumatological diseases), IgG4‐related disease, or other disease entities [4, 6–10]. CT and MRI cannot reliably distinguish these from RDD, as all may present with hyperdense masses on noncontrast CT with homogeneous enhancement, dural tails, and mixed T2 signals on postcontrast MRI. This is especially apparent in our case that the initial diagnosis and treatment targeted empirical diagnosis of meningioma. While both RDD and IgG4‐related diseases are diseases of the immune system, RDD is due to clonal proliferation of histiocytes, and IgG4‐related diseases are the result of immune dysregulation involving CD4‐positive T cells and plasma cells [7]. It is hard to distinguish these entities without biopsy [7, 11]. An important differential diagnostic consideration is other immune system diseases including granulomatous polyangiitis, other types of histiocytosis such as Langerhans cell histiocytosis or Erdheim–Chester disease, lymphoma (either primary CNS lymphoma or CNS involvement of systemic Hodgkin or non‐Hodgkin lymphoma), and melanoma [12]. While bacterial infections generally present acute symptoms, tuberculosis may also be considered in a patient presenting with subacute symptoms, which is why tuberculosis was not considered in our case. An indolent progression of 12 years is a unique situation to our case [6, 10]. Pathological diagnosis that includes immunohistochemistry is therefore critical to ensure diagnosis and optimal treatment, especially with the recent success of cobimetinib in treating RDD with KRAS and MEK alterations [5]. If RDD is suspected in the CNS but biopsy is not obtainable, imaging to look for systemic lesions is warranted, given the increased recognition of RDD and the availability of more efficacious treatments.

Cobimetinib is a MEK inhibitor that has been approved for use in melanoma and has recently been approved for RDD. Similar to the other MEK inhibitors and BRAF inhibitors, with which it is used in combination, cobimetinib may cause adverse effects including fever, fatigue, and liver and gastrointestinal toxicities in most cases [5, 13]. While there is limited evidence on the adverse effects attributed solely to cobimetinib due to its use in combination with other MEK or BRAF inhibitors, a meta‐analysis revealed that the patients receiving vemurafenib and cobimetinib had a higher incidence of adverse effects including cardiac myocardial infarction [13]. Despite the apparent relation, it is hard to associate our patient’s decline with the medication alone given the already poor prognosis.

Diagnosis of RDD is challenging due to its rarity and mimicry of more common diseases such as meningioma and neurosarcoidosis. While similar cases were previously reported, time from onset to histologically confirmed diagnosis of RDD was more than 12 years, the longest ever reported [4, 6, 14]. Because of the recent development of diagnostic guidelines and efficacious treatment for some patients with RDD and other histiocytoses, diagnostic workups for meningioma or neurosarcoidosis should include systemic imaging and biopsy if possible, especially when the location, response to therapy, and prognosis do not fit the considered etiology.

## 4. Declarations

Per our institutional policy, no consent for publication or approval from institutional review board is required for case reports. Authors declare no competing interests. There is no funding to declare. Data sharing is not applicable to this article as no datasets were generated or analyzed during the current study. Further details are available per request to the corresponding author.

## Author Contributions

Sam E. Gary and Serdar Akkol designed the study. Sam E. Gary, Serdar Akkol, Harshadayani J. Kumar, Christina Appin, and Xiaosi Han analyzed the patient’s data and contributed to the patient’s care. Christina Appin performed the histological examination of the meningeal biopsy. Sam E. Gary prepared the first draft of the manuscript.

## Funding

No funding was received for this manuscript.

## Disclosure

All authors reviewed and approved the final manuscript.

## Conflicts of Interest

The authors declare no conflicts of interest.

## Supporting Information

Additional supporting information can be found online in the Supporting Information section.

## Supporting information


**Supporting Information** CARE checklist, English‐2013‐4.

## Data Availability

The data that support the findings of this study are available on request from the corresponding author. The data are not publicly available due to privacy or ethical restrictions.
