# Supplementary material for: Presentation of Neurological Rosai–Dorfman Disease Over 12 Years: A Case Report
Source: Case Rep Neurol Med. 2026 Jul 26;2026:8933434. doi: 10.1155/crnm/8933434 (PMC13402897; doi:10.1155/crnm/8933434)
Supplement: Supplementary file 1 — Supporting Information CARE checklist, English‐2013‐4. [file CRNM-2026-8933434-s001.pdf]

CARE Checklist of information to include when writing a case report

CARE case report guidelines

(cc) BY-NC-ND

Topic	Item	Checklist item description	Reported on Line
Title	1	The diagnosis or intervention of primary focus followed by the words "case report"	1
Key Words	2	2 to 5 key words that identify diagnoses or interventions in this case report, including "case report"	45-46
Abstract (no references)	3a	Introduction: What is unique about this case and what does it add to the scientific literature?	23-29, 41-43
	3b	Main symptoms and/or important clinical findings	29-41
	3c	The main diagnoses, therapeutic interventions, and outcomes	29-41
	3d	Conclusion—What is the main "take-away" lesson(s) from this case?	41-43
Introduction	4	One or two paragraphs summarizing why this case is unique (**may include references**)	55-57
Patient Information	5a	De-identified patient specific information.	60-65
	5b	Primary concerns and symptoms of the patient.	72-78
	5c	Medical, family, and psycho-social history including relevant genetic information	62-71
	5d	Relevant past interventions with outcomes	62-69
Clinical Findings	6	Describe significant physical examination (PE) and important clinical findings.	72-78
Timeline	7	Historical and current information from this episode of care organized as a timeline	Figure 1
Diagnostic Assessment	8a	Diagnostic testing (such as PE, laboratory testing, imaging, surveys).	78-90; 92-102
	8b	Diagnostic challenges (such as access to testing, financial, or cultural)	n/a
	8c	Diagnosis (including other diagnoses considered)	79-82; 91-96; 98-102
	8d	Prognosis (such as staging in oncology) where applicable	n/a
Therapeutic Intervention	9a	Types of therapeutic intervention (such as pharmacologic, surgical, preventive, self-care)	103-109
	9b	Administration of therapeutic intervention (such as dosage, strength, duration)	103-105
	9c	Changes in therapeutic intervention (with rationale)	103-105
Follow-up and Outcomes	10a	Clinician and patient-assessed outcomes (if available)	n/a
	10b	Important follow-up diagnostic and other test results	n/a
	10c	Intervention adherence and tolerability (How was this assessed?)	105-106
	10d	Adverse and unanticipated events	105-109
Discussion	11a	A scientific discussion of the strengths AND limitations associated with this case report	112-122
	11b	Discussion of the relevant medical literature **with references**.	125-162
	11c	The scientific rationale for any conclusions (including assessment of possible causes)	125-143
	11d	The primary "take-away" lessons of this case report (without references) in a one paragraph conclusion	155-162
Patient Perspective	12	The patient should share their perspective in one to two paragraphs on the treatment(s) they received	n/a
Informed Consent	13	Did the patient give informed consent? Please provide if requested	Yes ☐ No ☒
